# Increased AGE-RAGE ratio in idiopathic pulmonary fibrosis

**DOI:** 10.1186/s12931-016-0460-2

**Published:** 2016-11-05

**Authors:** Carlos Machahua, Ana Montes-Worboys, Roger Llatjos, Ignacio Escobar, Jordi Dorca, Maria Molina-Molina, Vanesa Vicens-Zygmunt

**Affiliations:** 1 Pneumology Research Group, IDIBELL, University of Barcelona, Barcelona, Spain; 2 Department of Pneumology, Unit of Interstitial Lung Diseases, University Hospital of Bellvitge, Barcelona, Spain; 3 Research Network in Respiratory Diseases (CIBERES), Madrid, Spain; 4 Department of Pathology, University Hospital of Bellvitge, Barcelona, Spain; 5 Department of Thoracic Surgery, University Hospital of Bellvitge, Barcelona, Spain

**Keywords:** IPF, AGEs, RAGE, Extracellular matrix, 3D culture, Aging

## Abstract

**Background:**

The abnormal epithelial-mesenchymal restorative capacity in idiopathic pulmonary fibrosis (IPF) has been recently associated with an accelerated aging process as a key point for the altered wound healing. The advanced glycation end-products (AGEs) are the consequence of non-enzymatic reactions between lipid and protein with several oxidants in the aging process. The receptor for AGEs (RAGEs) has been implicated in the lung fibrotic process and the alveolar homeostasis. However, this AGE-RAGE aging pathway has been under-explored in IPF.

**Methods:**

Lung samples from 16 IPF and 9 control patients were obtained through surgical lung biopsy. Differences in AGEs and RAGE expression between both groups were evaluated by RT-PCR, Western blot and immunohistochemistry. The effect of AGEs on cell viability of primary lung fibrotic fibroblasts and alveolar epithelial cells was assessed. Cell transformation of fibrotic fibroblasts cultured into glycated matrices was evaluated in different experimental conditions.

**Results:**

Our study demonstrates an increase of AGEs together with a decrease of RAGEs in IPF lungs, compared with control samples. Two specific AGEs involved in aging, pentosidine and Nε-Carboxymethyl lysine, were significantly increased in IPF samples. The immunohistochemistry identified higher staining of AGEs related to extracellular matrix (ECM) proteins and the apical surface of the alveolar epithelial cells (AECs) surrounding fibroblast foci in fibrotic lungs. On the other hand, RAGE location was present at the cell membrane of AECs in control lungs, while it was almost missing in pulmonary fibrotic tissue. In addition, in vitro cultures showed that the effect of AGEs on cell viability was different for AECs and fibrotic fibroblasts. AGEs decreased cell viability in AECs, even at low concentration, while fibroblast viability was less affected. Furthermore, fibroblast to myofibroblast transformation could be enhanced by ECM glycation.

**Conclusions:**

All of these findings suggest a possible role of the increased ratio AGEs-RAGEs in IPF, which could be a relevant accelerating aging tissue reaction in the abnormal wound healing of the lung fibrotic process.

## Background

Idiopathic pulmonary fibrosis (IPF) is a chronic, progressive, and lethal interstitial lung disease of unknown cause [1]. IPF is characterized by an histologic pattern of usual interstitial pneumonia (UIP) which shows a heterogeneous distribution of dense parenchyma collagen deposition and active fibroblast foci alternating with areas of normal parenchyma [2]. An aberrant response of alveolar epithelial cells (AECs) to a repetitive damage that contributes to the loss of alveolar epithelial structures has been proposed in IPF physiopathology [3]. Thus, an imbalance between pro-fibrotic and anti-fibrotic factors leads to an uncontrolled extracellular matrix (ECM) formation that modifies the interstitial configuration [4]. This abnormal wound healing presents several hallmarks of accelerated aging [5].

Interestingly, the advanced glycation end-products (AGEs), oxidative non-enzymatic products derived from modified lipids, proteins or nucleic acids, have been implicated in some diseases related to an accelerated aging process [6–8], being proposed as markers of oxidative stress and aging [9]. Oxidants such as cigarette smoke and dietary AGEs would promote the glycation process, resulting in accelerated formation of endogenous AGEs, inducing cellular dysfunction and cell death [10]. Among dozens of AGEs described, pentosidine and Nε-Carboxymethyl lysine (CML) are the most studied. An accumulation of pentosidine has been reported in aged skin [11] and in some pulmonary diseases [12, 13], and a high presence of CML has been associated with cell response to oxidative stress [14].

The involvement of AGEs in promoting ECM protein modification and cross-linking is also remarkable [15, 16]. In this way, some studies have related a loss of tendon viscoelasticity when incubated with AGEs [17], and an increment of arterial stiffness driven by AGEs accumulation has been described in the aortic walls [18]. Moreover, a previous report from our group demonstrated stiffness changes in glycated 3D collagen matrices and fibroblast phenotypic transformation [19]. This type of in vitro model would mimic the cross-links and AGEs generation. Finally, AGEs can glycate some plasmatic proteins, such as globulins or albumin, changing their physicochemical properties [20, 21].

On the other hand, the effect of AGEs on cellular reactions has been suggested to be closely related to their receptor, RAGE [22, 23]. However, RAGE is a member of the immunoglobulin superfamily of receptors [24] that is highly expressed in type I AECs of healthy lungs [25], and has been related with the differentiation of type II to type I pneumocyte cells, lung development, re-epithelialization, and maintenance of epithelial adhesion to basement membrane [26–28]. RAGE can be located in the cell membrane (full-length RAGE, FL-RAGE), or soluble in the extracellular space (soluble RAGE, sRAGE), without the transmembrane and cytosolic domains [29]. Remarkably, different roles of these two isoforms of RAGE binding the AGEs have been suggested. AGE-bound FL-RAGE activate an inflammatory signaling pathway via the nuclear factor kappa B (NF-κB) that could increase RAGE expression, enhance pro-inflammatory mediators, modify oxidative balance, activate apoptosis, and block AGEs degradation [30]. Meanwhile, a preventive effect of their signaling pathway has been proposed when sRAGE joins AGEs [31]. Although the involvement of AGEs/RAGEs in the pulmonary fibrotic process remains unclear, some research groups have suggested a different pattern of expression, mainly for RAGEs [32–38]. Therefore, our study aims to evaluate the presence of AGEs in relation with RAGEs in IPF lungs, and the possible effect of AGEs on cell behavior.

## Methods

### Ethical statement

Control human lung samples were obtained from the distal area of 9 lobectomies of cancer, which showed a preserved pulmonary architecture without emphysema, infection or inflammation. IPF samples were obtained from 16 subjects who underwent surgical lung biopsy for diagnosis. The included IPF cases were discussed in the multidisciplinary Interstitial Lung Diseases Committee of the University Hospital of Bellvitge and were reported as histological UIP pattern in accordance with the American Thoracic Society/European Respiratory Society criteria [1]. All patients provided written informed consent and the study was approved by the Ethics Committee of our center (CEIC, ref. PR202/08).

### Patient characteristics

Both populations, controls and IPF, had similar demographic features without statistical differences: range of age (57.60 ± 7.47 and 63.61 ± 6.67 years, respectively) and male–female ratio in both groups (Table 1). Both groups included a high proportion of ex-smokers, and a few current smokers and non-smokers, with the duration of smoking cessation of 13.20 years ± 14.13 for the control group and 15.14 years ± 11.97 for IPF patients, showing no differences between both groups (*p* = 0.77). Furthermore, there were no differences between both groups in the average of pack-years (*p* = 0.94). The pulmonary functional test (PFT) showed significant differences between IPF and controls in forced vital capacity (FVC) and diffusing capacity for carbon monoxide (DLCO) (*p* < 0.01) (Table 1). Diabetic patients from controls and IPF were excluded from the study to avoid the AGEs formation inherent to diabetes mellitus disease.

**Table 1:** Group features

Patient characteristics	Control	IPF	*p*-value
Total	9	16	-
Gender (Male/Female)	7/2	13/3	-
Age	57.60 ± 7.47	63.61 ± 6.67	0.03
Smoking (Current/Former/Never)	2/5/2	4/10/2	-
Pack-years	24.91 ± 25.18	24.18 ± 26.81	0.94
Smoking cessation (years)	13.20 ± 14.13	15.14 ± 11.97	0.77
% FVC	99.36 ± 14.55	77.92 ± 15.05	0.00
% DLCO	89.43 ± 13.98	56.91 ± 18.44	0.00

Data expressed in mean ± SD. *FVC* forced vital capacity, *DLCO* diffusing capacity for carbon monoxide. Statistical differences were valued at *p*-value < 0.01

### Western blot analysis

Tissue samples were homogenized in radioimmunoprecipitation assay buffer (RIPA buffer) pH 7.60 with protease inhibitors (Sigma-Aldrich, USA) in Ultra-Turrax T25 basic (IKA®-Werke, Germany). Protein concentration was determined by BCA Protein Assay Kit (Thermo Fisher Scientific Inc., USA) and 30 μg of sample was loaded in Mini-PROTEAN® TGX™ precast 4–15 % polyacrylamide gel (Bio-Rad, USA) under reducing conditions. Gels were transferred onto nitrocellulose membrane in semi-dry Trans-Blot® Turbo™ Transfer System (Bio-Rad). After blocking with TBS-T 5 % BSA (Sigma-Aldrich), the membranes were incubated with rabbit polyclonal anti-AGEs 1:10000 (ab23722; Abcam, UK), rabbit polyclonal anti-CML 1:500 (ab27684; Abcam), mouse monoclonal anti-Pentosidine 1:1000 (KAL-KH012; Cosmo Bio Co., Japan) or mouse monoclonal anti-RAGE 1:1000 (ab54741; Abcam), and mouse monoclonal anti β-actin 1:2000 or mouse monoclonal anti α-tubulin 1:2500 (A1978 and T6199, respectively; Sigma-Aldrich) as a loading control during 1 h at room temperature (RT). Then the membranes were incubated with a secondary antibody: goat anti-IgG rabbit or anti-IgG mouse HRP conjugate (P0448 and P0447, respectively; Dako, Denmark) 1:1000 (for anti-AGE or anti-RAGE, respectively) in TBS-T during 1 h at RT. Membranes were washed in TBS-T 3 times for 5 min after the primary and secondary antibody incubation. Immunoblotting was detected by chemiluminescence in a LAS-3000 Imaging System (Fujifilm Holdings Corporation, Japan) with SuperSignal™ West Pico (Thermo Fisher Scientific), following the manufacturer’s recommendations. Densitometry was measured by MultiGauge image analyzer software (Fujifilm).

The antibodies used for global AGEs, pentosidine and CML, recognized any proteins which contain these regions in its structure, so the three bands observed in Western blot analysis (named AGEs/Pen/CML 1, 2 and 3) corresponding with three different molecular weights matched with three groups of AGE-modified proteins.

### Reverse transcriptase PCR

Total mRNA was extracted from IPF and control lung samples using TRIzol® reagent procedure (Invitrogen, UK) following the manufacturer’s instructions. Samples were quantified, and Reverse transcription reaction was done with 1 μg by iScript™ cDNA Synthesis Kit (Bio-Rad) in a thermal cycler (Bio-Rad). PCR was performed with DNA polymerase (Biotools B&M Labs, Spain) following the manufacturer’s advice; cDNA was mixed with RAGE primers (164 bp): sense 5′-CAGGACCAGGGAACCTACAG-3′ and antisense 5′-CATGTGTTGGGGGCTATCTT-3′; and β-actin primers (302 bp) were used as a housekeeping gene: sense 5′-GCACTCTTCCAGCCTTCCTTCC-3′ and antisense 5′-TGCTTGCTGATCCACATCTGCT-3′ (Sigma-Aldrich). Amplified samples ran in 2 % agarose gel electrophoresis with ethidium bromide. Then the gel was revealed within an UV chamber and images were developed in instant film to be digitalized afterwards.

### Immunohistochemistry

Control and IPF tissue samples were fixed using a 4 % formaldehyde solution in phosphate buffered saline (PBS) and embedded in paraffin. Then the samples were cut in 4 μm sections for the immunohistochemical procedures. Slides were deparaffinized and rehydrated, and then endogenous peroxidases were blocked by incubating samples with 3 % H_2_O_2_ for 10 min. AGE-BSA immunostaining was performed with polyclonal rabbit anti-AGEs antibody (Abcam), following the manufacturer’s instructions of Vectastain™ Elite Avidin-Biotin Complex Kit (ABC Kit) (Vector Laboratories, USA). Briefly, antigen retrieval was performed with boiling Tris-EDTA buffer pH 9. Subsequently, slides were blocked in an incubation solution (PBS, 0.2 % Triton X-100 and 0.2 % bovine gelatin) with 20 % of normal goat serum for 1 h at RT. Then, samples were incubated with primary antibody diluted 1:5000 in the incubation solution overnight at 4 °C. After that, slides were incubated in goat anti-rabbit IgG antibody 1:400 from Vectastain™ Kit during 1 h at RT. Later, slides were incubated with ABC Kit for 1 h at RT. Slides were washed with PBS 3 times for 5 min each, between incubations. A brown color was revealed with 3,3′-Diaminobenzidine tetrahydrochloride hydrate (Sigma-Aldrich) in PBS, and sections were counterstained with Harris’ haematoxylin (Casa Álvarez S.A., Spain) and cover-slipped with DPX (Merck Millipore, Germany).

RAGE immunostaining was made with mouse monoclonal anti-RAGE antibody (Abcam), following manufacturer’s instructions. In this case, antigen retrieval was made with boiling citrate buffer pH 6 (Dako). The antibody was diluted 1:200 in the incubation solution with 1 % of normal goat serum overnight at 4 °C. After that, slides were incubated in goat anti-mouse IgG2a HRP-conjugated antibody 1:200 (NB7516; Novus Biologicals, USA) diluted in the incubation solution with 1 % of normal goat serum. Revealing and counterstaining were performed following the same protocol explained above. A negative control was made by incubating control slides without primary antibody in all the immunohistochemical assays. The strong brown staining was considered as a positive signal. Images were evaluated by two expert pathologists blinded to the sample.

### Cell viability assay

In order to evaluate the AGEs effect in cell viability, primary fibroblasts from IPF lungs, A549 cell line (ATCC, Manassas VA, USA), and human airway epithelial (HAE) cell line (CRL-4011™, ATCC) were treated with AGE-BSA.

AGE-BSA was prepared following the protocol of Khan et al. [39]. Briefly, 50 mg/mL BSA (Sigma-Aldrich) was incubated with 1 M D-Ribose (Sigma-Aldrich) in PBS pH 7.4 for 20 days at 37 °C. In addition, BSA was incubated without D-Ribose as a control. After that, the solutions were dialyzed and filtered through 0.22 μm membranes (Merck Millipore) to remove D-Ribose debris; and then the presence of AGEs were checked and quantified by fluorescence (wavelength emission 440 nm/excitation 370 nm) and BCA Protein Assay Kit.

Fibrotic fibroblasts were isolated from IPF patients that underwent surgical lung biopsy and grew in DMEM (Gibco™, Thermo Fisher Scientific, USA) with 10 % FBS. A549 was cultured in F12K medium (Lonza, Switzerland) with 10 % FBS. HAE cell line was cultured in Bronchial Epithelial Cell Growth Medium (BEGM, Lonza).

Quick Cell Proliferation Colorimetric Assay Kit (MBL international, USA) was performed in 96-well plates following the manufacturers’ recommendations. Cells were seeded at 1 × 10^4^ cells/well and were incubated in medium with 2 % FBS for 24 h before the experiment. Then cells were treated with different concentrations of AGE-BSA for 3 h. A well without cells was assessed for all conditions as reference value. Reagent was added to each well for an additional 2 h and the plate was shaken and read in the Thermo Scientific Multiskan® EX (Thermo Fisher Scientific) at 450 nm, and absorbance was adjusted to the measurement in the reference value for each condition.

### 3D cell culture into glycated matrix

Three-dimensional culture based on glycated collagen matrix was performed to evaluate the behavior of fibrotic fibroblast cells under the influence of ECM cross-links and the consequent AGEs production. The 3D collagen matrices were produced using native type I collagen from bovine dermis at 4 mg/mL (Cosmo Bio Co., Japan), and were glycated adding ribose at 5 and 15 mM (Sigma-Aldrich), following a previously standardized protocol [19].

Fibroblasts were added and mixed with the collagen before polymerization of the gel. Cells were seeded into 96-well plates at 15 × 10^3^ cells/well. After matrix polymerization, a culture medium with a different concentration of ribose was added. Phenotype changes were evaluated by Western blot detection of alpha-smooth muscle actin (α-SMA) 1/500 (A5228; Sigma-Aldrich) on days 1, 7 and 14, following the procedure described above.

### Statistical analysis

Data of experimental groups were compared and analyzed with IBM SPSS Statistics 23 (IBM, USA). Differences between the two groups were analyzed by Student’s *t*-test or Mann–Whitney *U*-test when comparing two parametric or non-parametric samples, respectively. To evaluate the differences among experimental groups, one-way ANOVA was assessed. Results are expressed as means ± SD. The *p*-value < 0.05 (*) or < 0.01 (**) were considered statistically significant.

## Results

### Increased AGEs expression in IPF lungs

**Fig. 1:**
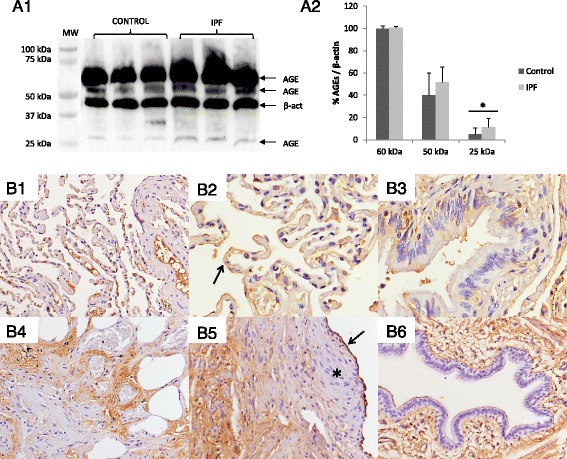
Presence of AGEs in human lung tissue from IPF patients (IPF) and control group (Control). **a1** The antibody used for global AGEs assessment recognized any proteins which contain a glycated region in structure; so the three bands seen (at 60, 50 and 25 kDa) in Western blot analysis correspond with 3 types of AGE-modified proteins. **a2** Although Anti-AGEs immunoblot did not show apparent differences between both groups, densitometry analysis showed statistical differences only in the band of 25 kDa and when the values of the three AGEs obtained were considered together. Presence of AGEs was represented as percentage in relation to loading control β-actin (β-act). Statistical analysis was performed using Student’s *t*-test (*p < 0.05). **b1** Parenchyma of lung control showed positive signal (*brown color*), mainly associated to lumen and endothelium of blood vessels and capillaries. **b2** Pneumocytes (*arrow*) showed a weak and discontinuous staining for anti-AGEs antibody. **b3** Ciliated epithelial cells of bronchus showed a blurred pattern of staining in their cytoplasm. **b4** Brown immunostaining associated to proteins from the ECM in IPF lungs. **b5** Reactive AECs (*arrow*) and ECM proteins showed immunoreactivity; contrary to fibroblast foci (*star*), where immunostaining was absent. **b6** Apical membrane of ciliated cells from bronchus and endothelium of blood vessels also showed staining. Micrographs recorded at 200X (**b1**, **4**, **5** and **6**) and 400X (**b2** and **3**) magnification

Western blot showed an increase of AGEs in IPF lungs, compared to control samples (Fig. 1, A1). Analyzing the three bands separately, the significant difference between both groups was achieved because of the highest expression of the 25 kDa band of AGEs in IPF samples (*p* < 0.05, Fig. 1, A2).

The immunohistochemical study of AGEs showed a different location pattern between both lung sections. In control pulmonary parenchyma, AGEs had a discontinuous pattern of staining, mainly in the endothelium of capillaries and veins, and there was a spotted staining in macrophages cytoplasm (Fig. 1, B1). Furthermore, pneumocytes (Fig. 1, B2) and bronchial epithelium (Fig. 1, B3) showed a weak staining for AGEs. Interestingly, IPF lung sections presented the strongest staining around the ECM proteins (Fig. 1, B4) and the apical surface of reactive AECs (Fig. 1, B5) and bronchial epithelium (Fig. 1, B6), while no signal was seen into the fibroblast foci.

**Fig. 2:**
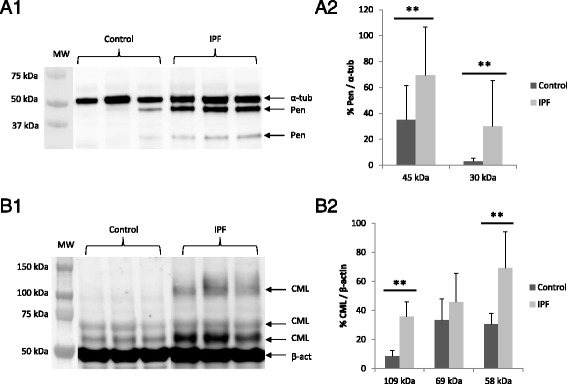
Pentosidine and CML expression by Western blot. **a1** Anti-pentosidine antibody used for Western blot assessment showed a dissimilar pattern of bands at 45 and 30 kDa. Two different pentosidine-modified proteins were recognized. **a2** Densitometry analyses showed a major presence of pentosidine in the IPF group compared with the control, for both bands. **b1** Anti-CML antibody showed several patterns of bands of CML-modified proteins: a high band in 109 kDa, and two bands in 69 and 58 kDa. CML at 109 and 58 kDa are stronger in IPF than control lungs. **b2** Densitometry analyses showed a major presence of CML-contained proteins in the IPF group in comparison to the control. Data were represented as percentage in relation to loading control α-tubulin (α-tub) and β-actin (β-act), respectively. Statistical analysis was performed using Mann–Whitney *U*-test and Student’s *t*-test, respectively (**p < 0.01)

Furthermore, two specific AGEs were individually evaluated; pentosidine and CML. Immunoblot analysis showed a high presence of pentosidine in IPF samples compared with controls (Fig. 2, A1). The two patterns of bands; 45 and 30 kDa, were significantly increased in IPF compared to control (*p* < 0.01, Fig. 2, A2). Meanwhile, Western blot assessment for CML-modified protein showed three patterns of bands: 109, 69 and 58 kDa (Fig. 2, B1). The highest band (109 kDa) and the lowest (58 kDa) were increased in IPF samples compared with control samples (*p* < 0.01, Fig. 2, B2).

### Decreased RAGE expression in IPF lungs

**Fig. 3:**
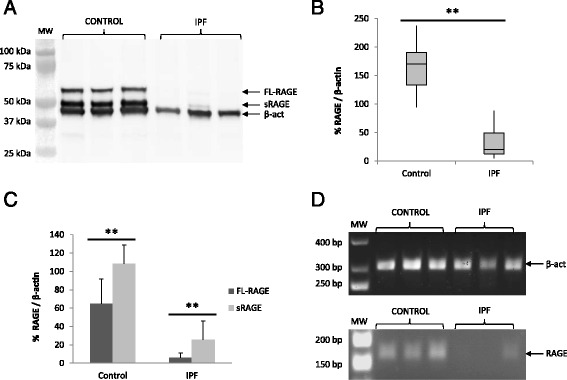
RAGE proteins and gene expression were decreased in IPF samples. **a** Immunoblot assay showed two strong RAGE bands in the control group: 56 (FL-RAGE) and 46 (sRAGE) kDa, respectively, whereas IPF samples showed a weak or absent signal for anti-RAGE antibody. **b** Densitometry analysis showed a higher amount of RAGE in the control group in respect to IPF. The total RAGE expression was represented in percentage in relation to β-actin (β-act) as loading control. **c** sRAGE/FL-RAGE ratio, for both groups. The sRAGE and FL-RAGE isoforms were both higher in the control group than in IPF and, interestingly, sRAGE was 1.67 and 4.17-fold higher than FL-RAGE, respectively. **d** RAGE (164 bp) and β-actin (302 bp) transcripts were amplified by RT-PCR. Control samples showed more RAGE gene expression than IPF samples. Statistical analysis was performed using Student’s *t*-test (**p < 0.01)

Lung homogenates were prepared to evaluate RAGE protein expression. Western blot analysis showed a strong RAGE expression in the control group; with two bands at 56 and 46 kDa, which correspond to the membrane or full-length RAGE (FL-RAGE) and the soluble RAGE (sRAGE) isoform, respectively (Fig. 3a), as described in the literature [40]. However, samples from IPF patients showed almost undetectable protein expression. Densitometry analysis showed a statistically significant decrease of RAGE protein expression in IPF compared to control lungs (*p* < 0.01, Fig. 3b). Analyzing both isoforms separately, we noticed that sRAGE isoform was 1.67 and 4.17-folds increased in relation to the FL-RAGE isoform, for both controls and IPF samples, respectively (Fig. 3c). Analyzing the gene expression of RAGE, a clear down-regulation was also observed in IPF lungs, compared with control lung homogenates (Fig. 3d).

**Fig. 4:**
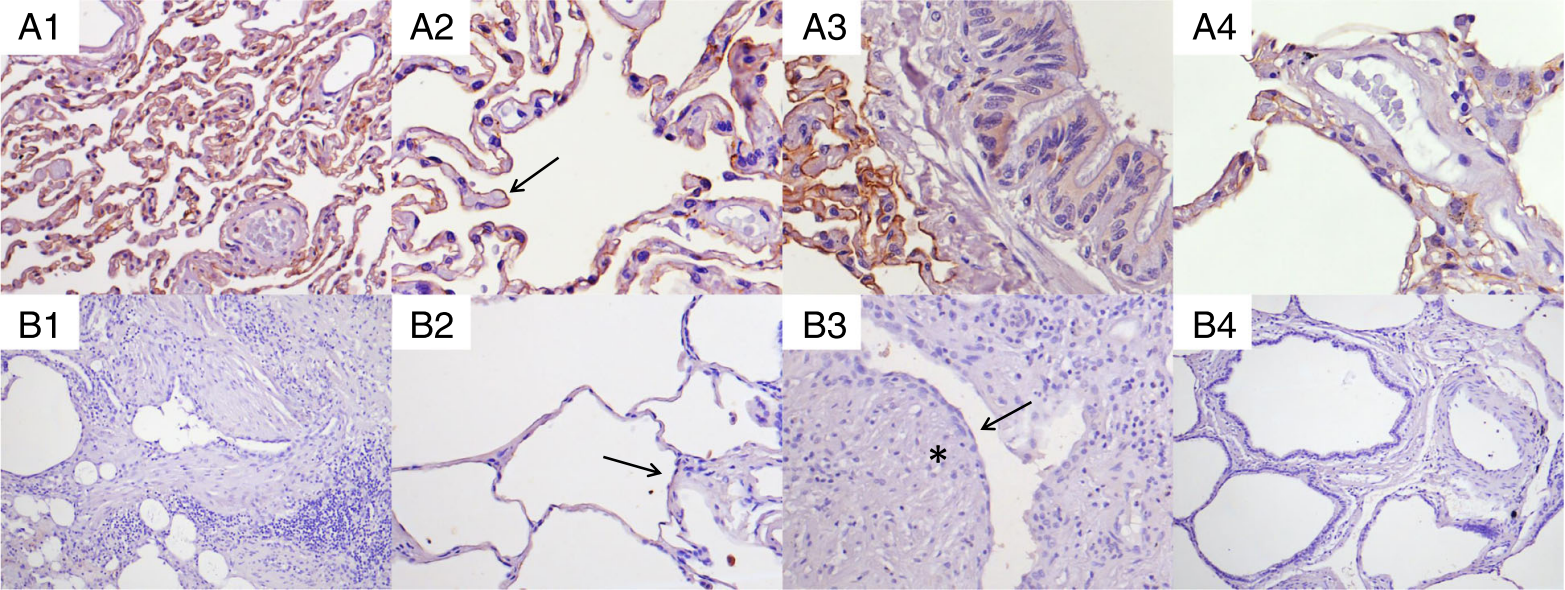
Immunostaining of RAGE in control (A) and IPF (B) lung sections. **a1** Parenchyma show a strong brown staining, focused in the alveolar epithelium. **a2** Pneumocytes show brown staining over its cell membrane (arrow), although there is a discontinuous pattern. **a3** Ciliated epithelial cells of bronchus show a weak staining pattern. **a4** Endothelium and smooth muscle of veins do not show brown staining. **b1** Areas of matrix deposition do not show immunoreactivity. **b2** Preserved parenchyma show a weak staining (*arrow*). **b3** Fibroblast foci (*star*) and AECs (*arrow*) that surround these fibroblast foci show almost undetectable immunostaining. **b4** No signal was considered in blood vessels and preserved bronchus. Micrographs were recorded at 100X (**b1** and **4**), 200X (**a1**, **b2** and **3**) and 400X magnification (**a2**, **3** and **4**)

To study the RAGE protein location in cells of human lung samples, an immunohistochemistry assessment was performed. Control lungs showed a strong positive staining for RAGE, mainly located in the cell membrane of AECs (Fig. 4, A1). Although the pattern of expression was not continuous around the epithelial membrane surface, we could distinguish the two sides of the alveolar septa (Fig. 4, A2). In addition, we also observed a weak immunostaining inside the cytoplasm of bronchial epithelial ciliated cells (Fig. 4, A3). Additionally, macrophages also exhibited a dotted brown staining. In contrast, the staining was absent in fibroblasts, and was not clearly defined in the endothelium and the smooth muscle of blood vessels (Fig. 4, A4). On the other hand, lung sample sections from IPF patients showed a heterogeneous immunostaining pattern. Areas with matrix deposition and restructured parenchyma did not show immunostaining (Fig. 4, B1), whereas preserved alveolar structures presented weak positive signal on the apical cell membrane surface of AECs (Fig. 4, B2). Meanwhile, the immunostaining for the RAGE antibody was totally absent in the hyperplasic AECs and fibroblast foci (Fig. 4, B3). Likewise, we did not find brown staining either smooth muscle cells or epithelial cells from vessels and bronchi (Fig. 4, B4).

**Fig. 5:**
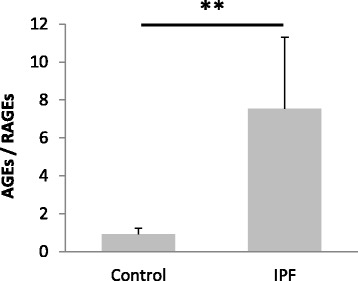
Ratio of the total AGEs/RAGEs. The proportion between total AGEs and RAGEs for each sample (IPF and controls) estimated after the semi-quantitative analysis (densitometry) from WB of AGEs and RAGE. Bars show the mean ± SD in both groups. The graph shows a considerable RAGE decrease in IPF samples, around 8.18-fold respect the control samples. Densitometry is measured by independent pair observers by using “MultiGauge image analyzer software Statistical analysis was performed using Student’s *t*-test (**p < 0.01)

Finally, we evaluated the AGEs/RAGEs ratio assuming a 1:1 equivalent relation between both semi-quantitative analyses. This ratio was 8.18-folds greater in IPF lungs compared to control samples, which demonstrate a significant imbalance of the AGEs/RAGEs since both increase of AGEs and decrease of RAGEs in IPF lungs (Fig. 5).

### Effect of AGEs in cell viability and phenotype

**Fig. 6:**
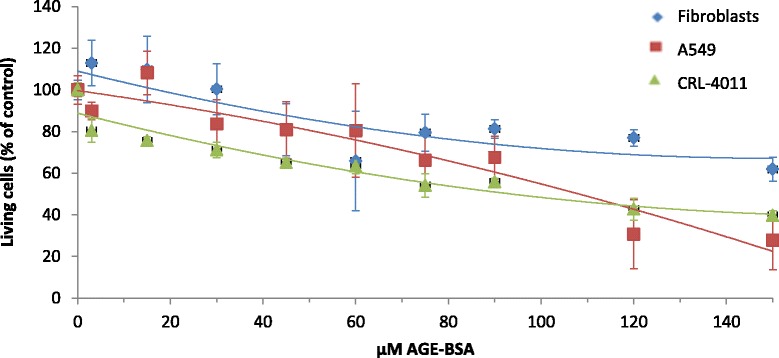
Cell viability assay of AGE-BSA in fibroblast, A549 cell line and human airway epithelial (HAE) cell line. Line tendency shows the response of fibroblast (*blue line*), A549 cell line (*red line*) and HAE cell line (*green line*) to AGE-BSA (0 to 150 μM) after 3 h of incubation. Data is expressed as mean ± SD. MTT was assessed in triplicate

Cell viability assay showed a different dose–response curve to AGE-BSA for the cell types. A549 AECs showed a defined slope (Fig. 6, red line) and the cell viability decreased at all concentrations, with a higher effect at the maximum concentration tested (150 μM) that associated less than 30 % viability. Meanwhile, HAE cells (Fig. 6, green line) had also shown a dose-dependent decrease of cell viability with AGE-BSA. By contrast, human lung fibrotic fibroblasts showed a line tendency with a smooth slope (Fig. 6, blue line), reaching 60 % of living cells at the highest AGE-BSA dose.

**Fig. 7:**
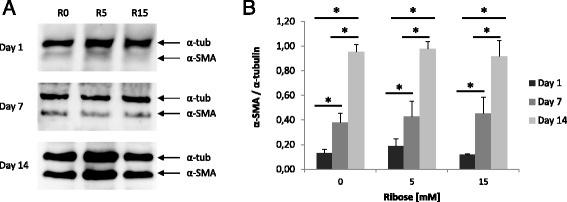
Protein expression of α-SMA from fibroblast in glycated matrix. **a** Fibroblasts were cultured within 3D collagen matrix and collected at days 1, 7 and 14 to study a possible phenotypic profile change. Western blot showed an increment in all ribose concentrations (R0, 5 and 15 mM) by day. **b** Densitometry did not find significant differences between control and R5 and 15 mM. The experiment was assessed in triplicate and represented as percentage in relation to loading control α-tubulin (α-tub). Statistical analysis was performed using Student’s *t*-test (*p < 0.05)

In addition, fibrotic fibroblasts were cultured into non-enzymatic glycated collagen matrix to evaluate their phenotype changes growing in an enriched AGEs ECM microenvironment. Protein expression of α-SMA showed a significant increment in all conditions from day 7 (Fig. 7a). Interestingly, Western blot analysis did not show significant differences between glycosylated conditions (with and without ribose) (Fig. 7b).

## Discussion

In the last few years, IPF has been proposed as a result of an accelerated aging process of the lung [5]. Some findings support ECM as a target of oxidative stress in the lung and the subsequently produced AGEs, which are biomarkers of an in vivo aging process that may promote fibrogenesis [9, 41]. In this line, our results demonstrate that AGEs are increased in IPF samples at the same time that RAGEs are decreased. Specifically, two well-known AGEs, CML and pentosidine-modified proteins, which have been related to aging, tissue stiffness and AECs apoptosis [11, 14, 18] are significantly overexpressed in IPF samples. Some pathogenic features of IPF may be implicated in the AGEs formation; collagen deposition, aging, oxidative compounds derived from smoking, dust or diet, and decreases of soluble RAGE are processes that could enhance the accumulation of AGEs in lung fibrosis [15].

At the same time, the higher amount of AGEs formation could influence the perpetuation of the fibrogenic process. In vitro studies have shown that AGEs could induce cell toxicity and death [39], delaying wound healing in epithelial cells [42]; whereas enhancing collagen and transforming growth factor (TGF)-β1 synthesis [43]. Additionally, AGEs induce epithelial-mesenchymal transition (EMT) in epithelial cells from rat kidney [44], although no EMT effect has been found in type II AECs from the rat lung [45]. Actually, some studies have even suggested that blocking the AGEs might attenuate pulmonary fibrosis [46]. However, there is scarce information about the effect of AGEs in human AECs and fibroblasts from lung parenchyma. In the present study, we demonstrated, as an initial approach, that alveolar type II epithelial cell line viability (A549) and HAE cell line were more sensitive to the presence of AGEs, while fibrotic fibroblasts had a low response to high dosage of AGEs; suggesting a different effect of AGEs, depending on cell type. Although the A549 cell lines may be suitable for initial exploratory IPF studies, data derived from such cells must acknowledge the limitations associated with these tools [47].

In addition, immunohistochemical results showed increased AGEs associated with the hyperplasic AECs, which suggest that AGEs could induce pro-fibrotic effects such as loss of epithelium. On the other hand, our results indicate the presence of myofibroblast transformation from fibrotic fibroblasts, in part because of the presence of AGEs at the surrounding ECM. Previous work of our group has demonstrated that 3D collagen matrices generated under glycation present an increase of AGEs, which is time-dependent and appears 5 days after glycation begins [19]. The AGEs formation is not only dependent on ribose concentration, but also on the presence of serum and glucose in the media, which would explain the fact that fibroblast-myofibroblast transformation appears in glycated 3D matrices, with and without ribose addition. However, a potential limitation for the interpretation of this observation is that other non-controlled collateral collagen cross-link reactions described at the glycated process of this in vitro model and not associated with AGEs formation could also influence in the myofibroblast transformation.

On the other hand, since a higher presence of RAGE has been found in healthy pulmonary tissue, in comparison to other organs, there are many studies that support its outstanding role in pulmonary homeostasis [25]. Our results demonstrated that the protein expression of RAGE (FL-RAGE and sRAGE) was decreased in all fibrotic lungs compared with control lungs. According to this observation, Quiesser et al. suggested an important function of RAGE in cellular adhesion and spreading to the basal membrane, playing a structural role in the maintenance of the alveolar epithelium [32]. It has also been demonstrated that RAGE stimulates elastin expression and plays a supporting role in respiratory mechanics [48]. Furthermore, RAGE may belong to a family of cell adhesion molecules [49], making links with basal lamina components such as type IV collagen or laminin [28, 50]. Thus, hypothetically, this RAGE down-regulation noticed in IPF lung samples could reduce the interaction of AECs to ECM and facilitate the basal membrane disruption, resulting in the occupation of alveolar spaces. Additionally, our results showed that the soluble isoform, sRAGE, is the greatest RAGE variant in the control lungs, with a sRAGE/FL-RAGE ratio of 1.67 in agreement with other studies performed in human lungs [51]. Interestingly, it has been described that only 7 % of RAGE transcript encodes for the main alternative splicing soluble variant [29], so a greater part of sRAGE might come from the cleavage of FL-RAGE by metalloproteinases [52], which are overexpressed in lung tissue and BAL fluids from IPF patients [53]. Consequently, it could cause the loss of joins between the type I AECs and the basement membrane, avoiding the normal re-epithelialization in response to damage [54]. In support of this, our results showed a sRAGE/FL-RAGE ratio in IPF lungs of 4.17, 2.5 fold increased in respect to the control lungs, in the possible context of the MMPs’ raising in the fibrotic process.

Regarding our immunohistochemical analysis of RAGEs, a predominance location around the cell membrane of AECs was observed in control lung tissues, as was previously described [55]. In contrast, RAGE was totally absent in the hyperplasic AECs and fibroblast foci from IPF lungs. Although this decrease of RAGE in fibrotic lung samples could be explained by the loss of AECs, some studies suggest that RAGE might be decreased from the beginning of AECs damage [33, 56]. Likewise, in our study we have observed that the loss of RAGE was not restricted only to protein expression, but also to the genetic expression. Hence, RAGE reduction is not only associated with Type I AECs’ disappearance as a consequence of tissue remodeling, but also the areas of preserved parenchyma show a decreased RAGE expression.

Although solid evidence demonstrates a complex function of RAGE in the lung, the implication grade and the role in lung physiology is still under discussion. Furthermore, the decrease of RAGE expression in IPF, especially sRAGE, suggests a potential utility to be tested in the future as biomarker [57] and therapeutic target [31, 57–59].

## Conclusion

In conclusion, our results suggest the implication of AGEs accumulation beside the decrease of RAGEs in the altered wound healing of IPF. The current findings demonstrate that the fibrotic lung presents an AGEs/RAGEs imbalance, which could associate oxidative damage in the accelerated aging process. Therefore, the advance in the knowledge of AGEs-RAGE function in pulmonary fibrosis, due to the particularities that RAGE presents in the lung homeostasis and the implication of AGEs in degenerative diseases and aging, would be essential to explore through this possible new pathway.

## Abbreviations

AECs: Alveolar epithelial cells
AGEs: Advanced glycation end-products
CML: Nε-Carboxymethyl lysine
DLCO: Diffusing capacity for carbon monoxide
ECM: Extracellular matrix
EMT: Epithelial-mesenchymal transition
FL-RAGE: Full-length RAGE
FVC: Forced vital capacity
HAE: Human airway epithelial
IPF: Idiopathic pulmonary fibrosis
NF-κB: Nuclear factor kappa B
PFT: Pulmonary functional test
RAGE: Receptor for AGEs
sRAGE: Soluble RAGE
TGF-β1: Transforming growth factor beta 1
UIP: Usual interstitial pneumonia
α-SMA: Alpha-smooth muscle actin
